# Supplementary material for: Interleukin-17, a salivary biomarker for COVID-19 severity
Source: PLoS One. 2022 Sep 22;17(9):e0274841. doi: 10.1371/journal.pone.0274841 (PMC9498944; doi:10.1371/journal.pone.0274841)
Supplement: S1 Fig — (A) IL-17 mRNA levels in nasopharyngeal swabs of COVID-19 patients compared to healthy controls (n = 430 COVID-19 patients vs n = 54 healthy controls; GSE152075). (B) IL-17 mRNA levels in lung autopsies of COVID-19 patients compared to healthy controls (n = 17 SARS-CoV-2 infected lung vs. n = 5 healthy lung biopsies; GSE150316). Statistical test: Unpaired t-test or Mann-Whitney U test, depending on the skewness of the data, * P<0.05. (PDF) [file pone.0274841.s001.pdf]

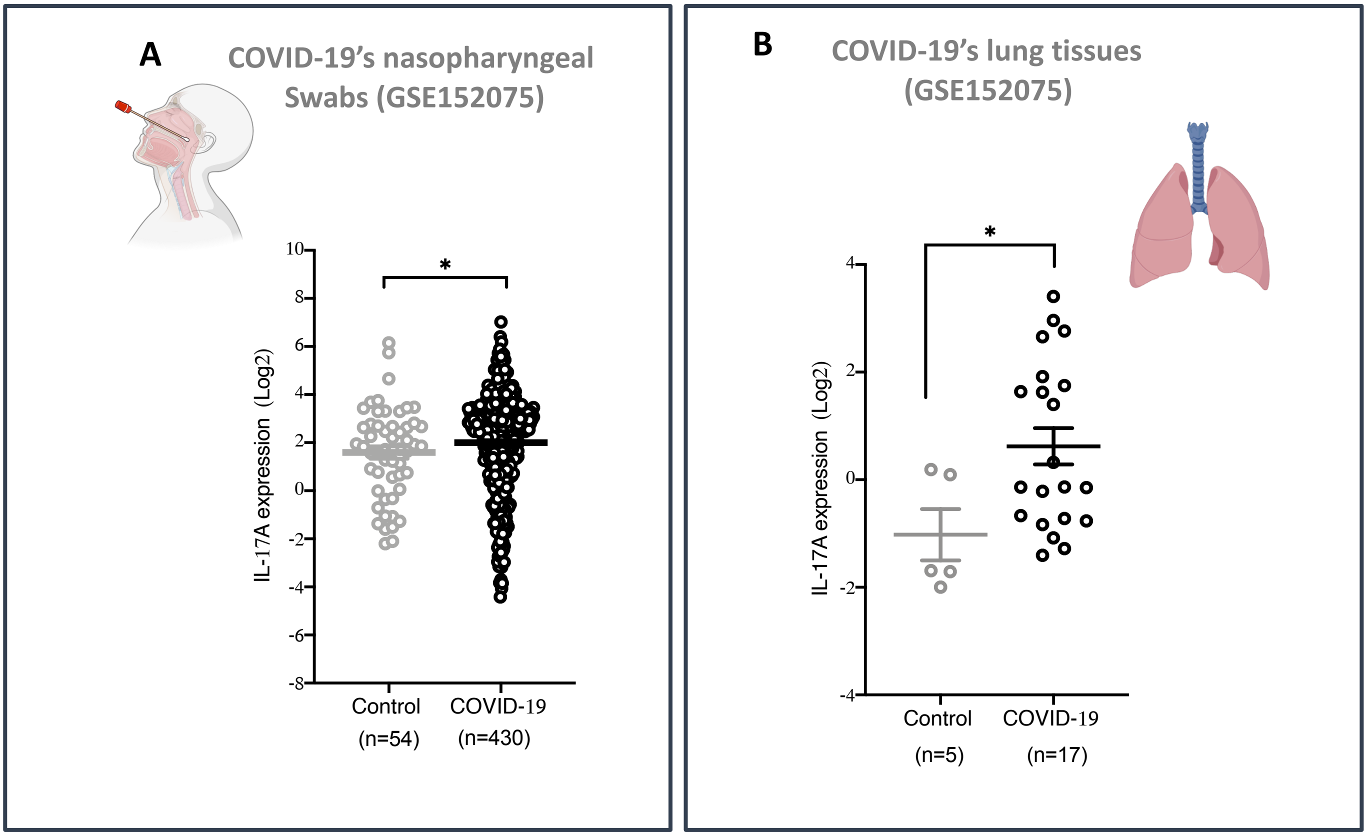

Supplementary Figure 1. Increased IL-17 gene expression levels in lung and nasopharyngeal swabs of COVID-19 patients. (A) IL-17 mRNA levels in nasopharyngeal swabs of COVID-19 patients compared to healthy controls (n=430 COVID-19 patients vs n=54 healthy controls; GSE152075). (B) IL-17 mRNA levels in lung autopsies of COVID-19 patients compared to healthy controls (n=17 SARS-CoV-2 infected lung vs. n=5 healthy lung biopsies; GSE150316). Two-way comparison was done using unpaired t-test or Mann-Whitney U test, depending on the skewness of the data, * P<0.05.
