# Supplementary material for: Interleukin-17, a salivary biomarker for COVID-19 severity
Source: PLoS One. 2022 Sep 22;17(9):e0274841. doi: 10.1371/journal.pone.0274841 (PMC9498944; doi:10.1371/journal.pone.0274841)
Supplement: S2 Fig — Data show that IL-17 in these COVID-19’s nasopharyngeal swabs positively correlate with levels of IL-1β, TNFα, IL-6, IL-8 and CCL2, but not IFNγ. Statistical test: Pearson’s coefficient test with two-tailed p-value <0.05 considered significant. (PDF) [file pone.0274841.s002.pdf]

COVID-19's nasopharyngeal swabs (GSE152075)

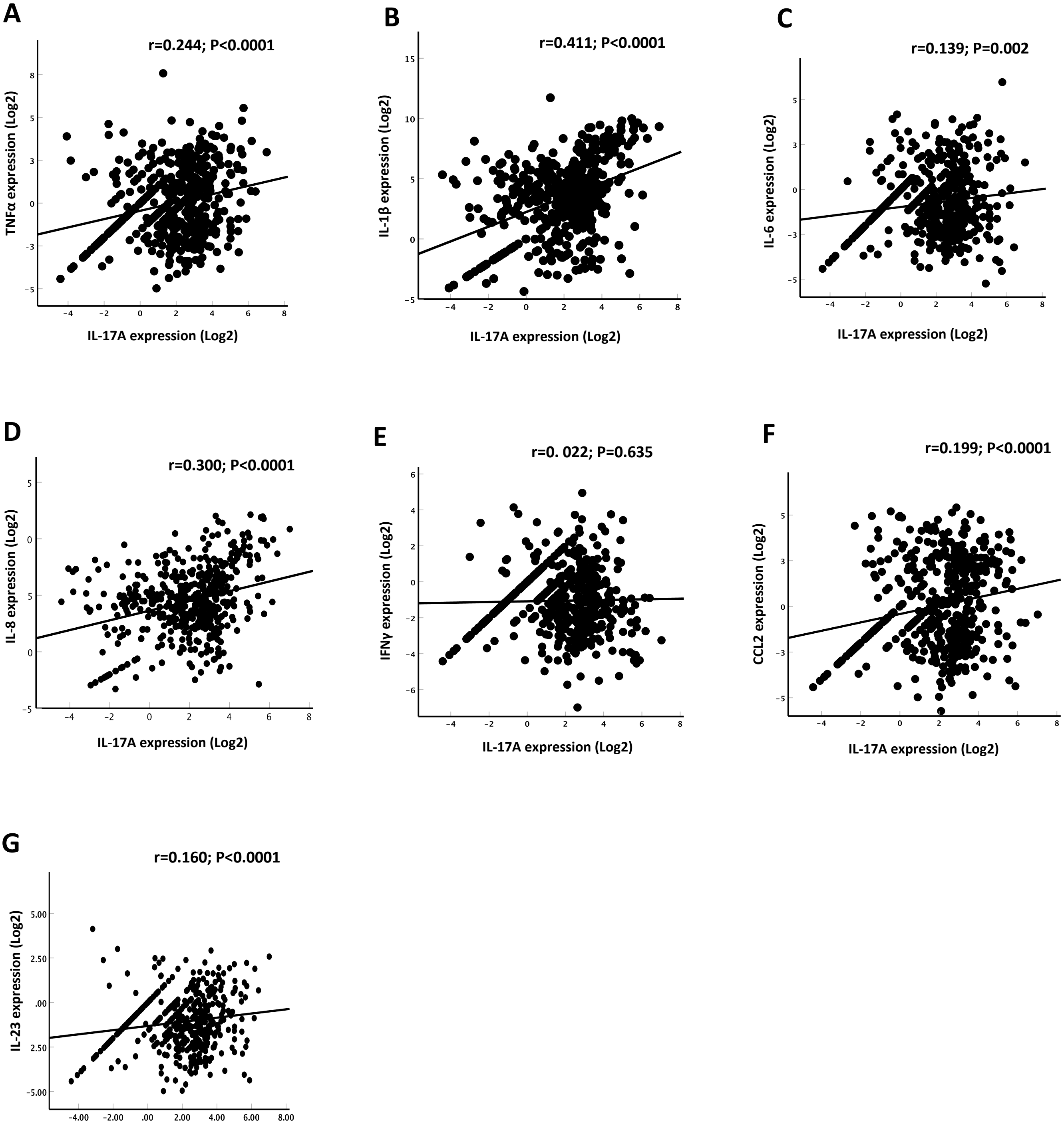

Supplementary Figure 2. Correlation between IL-17 expression level and Th-17 signaling related cytokines/chemokines such as TNFα, IL-1β, IFNγ, IL-6, neutrophils chemoattractant IL-8, and monocytes chemoattractant, CCL2 in nasopharyngeal swabs of COVID-19 patients (n=430 COVID-19 patients; GSE152075). Data show that IL-17 in these COVID-19's nasopharyngeal swabs positively correlate with levels of IL-1β, TNFα, IL-6, IL-8 and CCL2, but not IFNγ. Correlation test was done using the Pearson's coefficient test with two-tailed p-value <0.05 considered significant.
