# Supplementary material for: Interleukin-17, a salivary biomarker for COVID-19 severity
Source: PLoS One. 2022 Sep 22;17(9):e0274841. doi: 10.1371/journal.pone.0274841 (PMC9498944; doi:10.1371/journal.pone.0274841)
Supplement: S3 Fig — Data show that IL-17 level in COVID-19’ lung autopsies positively correlate with levels of TNFα, IL-1β, IFNγ, IL-6, IL-8 and CCL2, but not CCL2. Statistical test: Pearson’s coefficient test with two-tailed p-value <0.05 considered significant. (PDF) [file pone.0274841.s003.pdf]

COVID-19’s lung tissues (GSE150316)

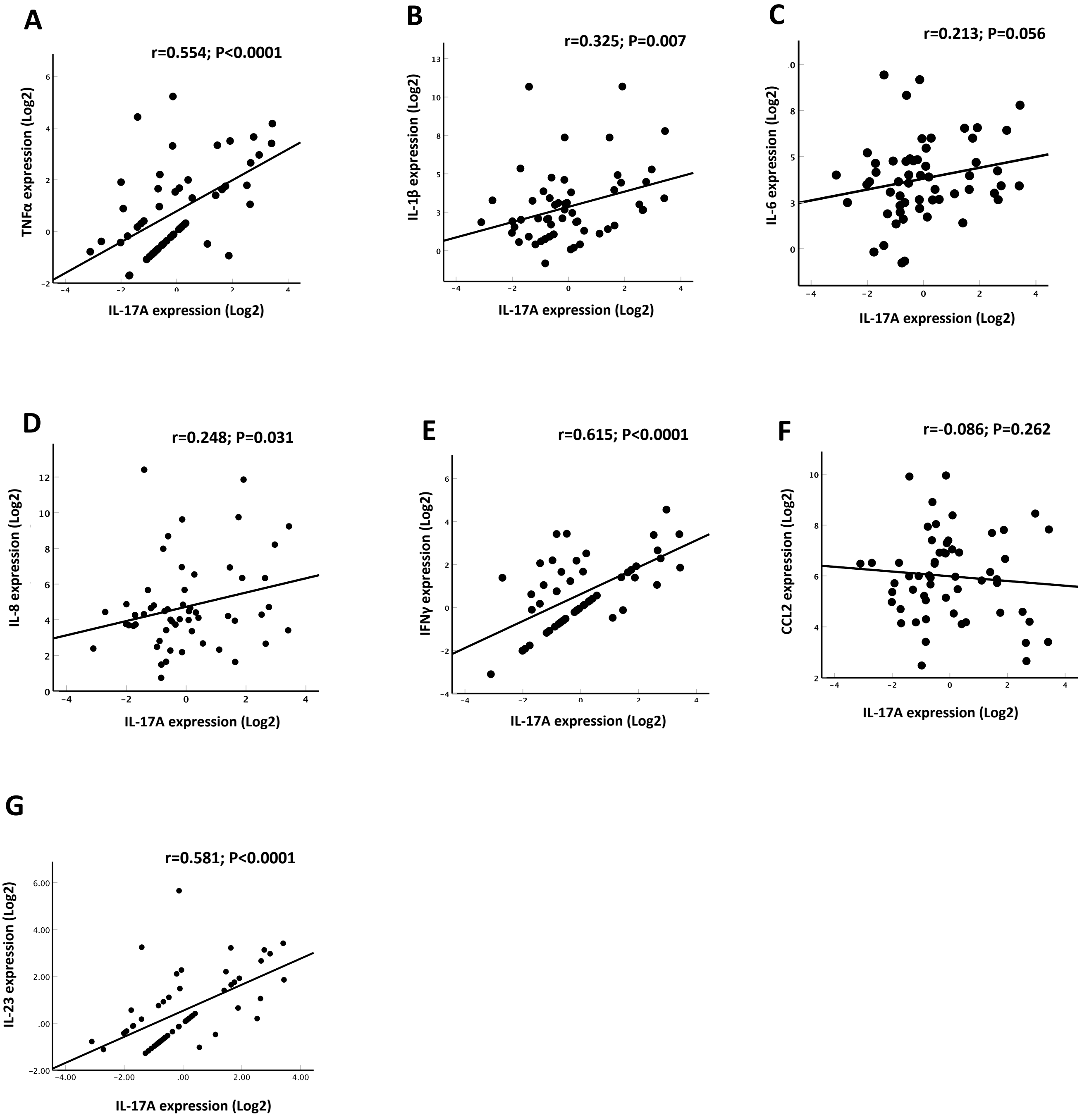

Supplementary Figure 3. Correlation between IL-17 expression level and Th-17 signaling related cytokines/chemokines such as TNFα, IL-1β, IFNγ, IL-6, IL-8, and CCL2 in lung autopsies of COVID-19 patients (n=17 SARS-CoV-2 infected lung tissues; GSE150316). . Data show that IL-17 level in COVID-19’ lung autopsies positively correlate with levels of TNFα, IL-1β, IFNγ, IL-6, IL-8 and CCL2, but not CCL2. Correlation test was done using the Pearson’s coefficient test with two-tailed p-value <0.05 considered significant.
